# Dispersive micro solid phase extraction of cadmium on MIL-53(Al)@BaTiO_3_ nanocomposite from seafood samples

**DOI:** 10.55730/1300-0527.3733

**Published:** 2025-04-15

**Authors:** Mustafa SOYLAK, Eda BORA, Furkan UZCAN

**Affiliations:** 1 Department of Chemistry, Faculty of Sciences, Erciyes University, Kayseri, Turkiye; 2 Technology Research and Application Center (TAUM), Erciyes University, Kayseri, Turkiye; 3 Turkish Academy of Sciences (TUBA), Ankara, Turkiye

**Keywords:** Cadmium, seafoods, micro solid phase extraction, nanocomposite

## Abstract

This study’s main goal was to produce a user-friendly dispersive micro solid phase extraction (dmSPE) technique with a MIL-53(Al)@BaTiO_3_ nanocomposite for the extraction and preconcentration of cadmium (Cd) in various seafood matrices, followed by using high-resolution continuum source flame atomic absorption spectrometry (HR-CS-FAAS). The MIL-53(Al)@BaTiO_3_ nanocomposite was synthesized and characterized using a range of techniques, including Fourier-transform infrared spectroscopy, field emission scanning electron microscopy, scanning transmission electron microscopy, X-ray diffraction, and Brunauer-Emmett-Teller analysis. The dmSPE technique involved the dispersion of the MIL-53(Al)@BaTiO_3_ material in the sample solution, followed by its separation from the sample matrix. The optimized method exhibited a linear range of 3.6–250 μg L^−1^, a limit of detection (LOD) of 1.2 μg L^−1^, and a preconcentration factor of 80. Two different certified reference materials were used to ensure the validation of developed method. The method was applied to different seafood samples.

## 1. Introduction

Heavy metals, also known as permanent pollutants, are trace metals that have a density of at least five times greater than water. Due to their robustness and inability to break down in the body, they are bioaccumulative and can be harmful when they make their way up the food chain to humans [1–3]. Moreover, these metals can have detrimental effects on aquatic life and the environment. Heavy metals are mostly nonessential elements that can be highly toxic to the human body [4,5]. They lack any physiological function and can enter the body through skin absorption, inhalation, and ingestion. These metals are frequently discharged into the environment by a variety of channels, such as food, drink, air, and items and chemicals produced by humans. Once released, they can easily permeate our bodies, potentially causing significant harm [6–9].

Cadmium is a dangerous heavy metal that can be extremely toxic, even in small amounts. It has the potential to build up in organisms and ecosystems, and once it enters the human body, it can remain there for an extended period ranging from 10 to 33 years. The World Health Organization recommends that no more than 0.4–0.5 mg of cadmium be consumed per week, or 0.057–0.071 mg per day to prevent health damage. Prolonged exposure to cadmium can also lead to renal damage. As a result, cadmium is considered a priority pollutant for monitoring by most countries and international organizations [10–13].

In the trace metal determination, it is commonly acknowledged that a separation-preconcentration procedure is crucial for enhancing the sensitivity and selectivity of the determination [14,15]. Dispersive micro-solid-phase extraction (dmSPE) has been the focus of recently published studies as an innovative method for separation and preconcentration. This approach uses a minimal amount of adsorbent that is dispersed in the sample solution. Because of its simplicity of use, quickness, minimal need for organic solvents, and versatility with various detection methods, dmSPE has attracted a lot of interest [16–18].

Metal ions and organic linkers self-assemble via coordination bonds to form metal–organic frameworks (MOFs), hybrid inorganic–organic microporous crystalline materials. With regard to their large surface area, consistently formed cavities, and chemical and thermal durability, MOFs are excellent adsorbents that are frequently utilized in sample pretreatment [19]. Because of its exceptional water stability and special breathing action, which adjusts the pore size to the guest species without causing crystallinity loss or bond breakage, MIL-53(Al) is a desirable MOF. MIL-53(Al) is appropriate for application in liquid-phase separation and adsorption because of these remarkable features [20–22].

BaTiO_3_ is acknowledged as one of the most versatile materials of the modern era. As a consequence of its superior properties, piezoelectric and dielectric capabilities, together with the appropriate temperature factor impact, have led to its use in a wide range of applications, including supercapacitors, piezoelectric devices, thermistors, ferroelectric devices [23].

In the present study, MIL-53(Al)@BaTiO_3_ was synthesized and characterized. It was used as Nano sorbents in the dmSPE method for Cd(II) in seafood samples from Türkiye. The analytical parameters like pH, adsorbent amount, eluent etc. were optimized for the quantitative recoveries of Cd(II).

## 2. Experimental

### 2.1. Chemicals and reagents

All the chemicals used in the experiments were of high quality. The study utilized ultra-pure water from Millipore Milli-Q Direct 16, which has a resistivity of 18.2 MΩ cm. The Labsert company in Mersin, Türkiye, provided a stock solution of Cadmium (1000 mg L^−1^). The stock solution of Cd(II) was diluted with ultra-pure water to produce the working and calibration solutions. Zinc nitrate hexahydrate was purchased from Carlo Erba Milano, Italy. The sodium phosphate dibasic heptahydrate, sodium phosphate monobasic monohydrate, sodium acetate, acetic acid, and the other chemicals were obtained from Merck in Darmstadt, Germany. Sample solutions were produced in the pH ranges of phosphate buffer solutions (6.0–8.0) and acetate buffer solutions (4.0–5.0). SPS-WW2 and NIST CRM 1577b were purchased from LGC Standards and the National Institute of Standards and Technology (US), respectively.

### 2.2. Instruments

The ContrAA 800 spectrometer (HR-CS-FAAS, Analytik Jena) was used to perform atomic absorption measurements. It has a 300W xenon short-arc lamp in hotspot mode as a radiation source, along with a high-resolution double monochromator and a linear charge-coupled device (CCD) array detector with 588 pixels. The measurements were carried out using an air-acetylene flame with and oxidizer fuel flow rates of 400 L h^−1^ and 65 L h^−1^, respectively. The wavelength and bandwidth values for cadmium were set to 228.8 nm and 0.1 nm, respectively.

Weighing of compounds was performed with the use of the Radwag AS220 analytical balance from Radwag (Poland), which has a sensitivity of 0.1 mg. pH measurements were obtained with a digital pH meter model WTW 3110 from WTW GmbH, Germany, which was calibrated using buffer calibration solutions. The Rotofix 32A centrifuge from Hettich, Germany, was utilized during the study. Fisons WhirliMixer™ (England), was implemented to promote interaction between the sorbent and cadmium ions. FT-IR 400 Perkin-Elmer spectrometer (USA) was utilized. Additionally, for FE-SEM, and STEM analyses, the Zeiss Gemini 500 field emission scanning electron microscope (Germany) was utilized. The Gemini Micromeritics VII surface analysis method was utilized to examine and describe the properties of the composite. A diffractometer with monochromatic Cu-Kα radiation was employed for the X-ray diffraction (XRD) patterns of the adsorbent. Lastly, Ethos Lean microwave oven (Milestone, Italy) was used for the digestion processes.

### 2.3. Synthesis of MIL-53(Al)@BaTiO3

The provided procedure outlines the synthesis of MIL-53(Al) followed by the preparation of a composite material. To initiate the synthesis of MIL-53(Al), 1.3 g of Al(NO_3_)_3_. 9 H_2_O and 0.58 g of C_6_H_4_(CO_2_H)_2_ are weighed and mixed in a beaker containing a solvent blend consisting of 22.5 mL DMF and 7.5 mL ultra-pure water. After stirring for 30 min, the mixture is subjected to microwave irradiation at 200 W for 3 h. Subsequently, the resulting compound undergoes washing twice with DMF and ethanol before being dried at 120 °C [23].

For the synthesis of the MIL-53(Al)@BaTiO_3_, 1 mmol of barium nitrate is dissolved in 5 mL of acetic acid in a separate vessel. To this solution, 0.2 g of microwave synthesized MIL-53(Al) is stirred vigorously to create a homogeneous suspension. Concurrently, 1 mmol of titanium(IV) butoxide is dissolved in 5 mL of ethanol, then incorporated into the mixture to form a combined solution. The pH of this mixture is adjusted to approximately 14 using a 5 mol L^−1^ sodium hydroxide solution prior to its transfer into a Teflon-lined autoclave [24]. The autoclave is heated to 180 °C and maintained at this temperature for 16 h. Following the heating process, the autoclave is allowed to naturally cool down to 25 °C. The product is collected, washed with ultra-pure water until neutralization is achieved, and finally dried at 80 °C for 12 h [24,25].

### 2.4. Dispersive micro-solid-phase extraction procedure

Cylindrical centrifuge tubes of 50 mL were utilized to prepare a solution of Cd (II) with a concentration of 2.5 μg L^−1^. The sample solution pH was adjusted to 5.0 using acetate buffer, and subsequently, 30 mg of MIL-53(Al)@BaTiO_3_ was transferred to allow the sorption of Cd(II). To improve, effectiveness of extraction, the tubes were vortexed for 90 s. Following the adsorption, the composite was separated from the sample through 3 min of centrifugation. Subsequently, the analyte ions were sorbed to the MIL-53(Al)@BaTiO_3_ nanocomposite were released using 0.5 mL of 1.5 mol L^−1^ HNO_3_ and 0.5 min of vortex. To determine the analyte amounts, the eluent was fed into the HR-CS-FAAS three times.

### 2.5. Applications

Commercial typical food samples (shrimp, mussel, and tuna fish samples) purchased from the local markets near Kayseri were used to further evaluate the method’s reliability. When purchased from the supermarket, shrimp, mussel, and tuna fish samples were all deceased and already packaged in special boxes. In compliance with the instructions of the microwave system, NIST CRM 1577b, and a 0.25 g sample of shrimp, mussel (without shell), and tuna fish were digested separately in a closed microwave digestion unit, utilizing five mL of conc. HNO_3_ and one mL of H_2_O_2_. The digested samples were subsequently diluted with ultra-pure water to reach a total volume of 40 mL. Then the procedure given in Section 2.4 was applied. SPS-WW2 certified reference material was employed to procedure given in Section 2.4 after appropriate dilutions.

## 3. Results and discussion

### 3.1. Characterization of MIL-53(Al)@BaTiO3

**Figure 1:**
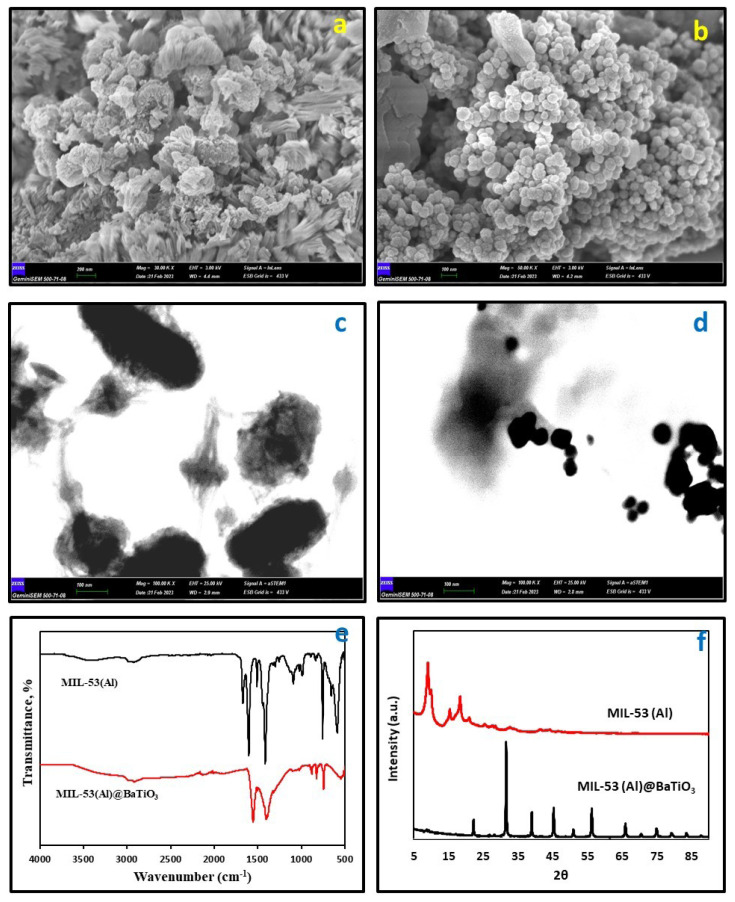
Characterization of the MIL-53(Al)@BaTiO_3_ nanocomposite: (a, b) Field Emission Scanning Electron Microscopy (FE-SEM) and (c, d) Scanning Transmission Electron Microscopy (STEM) images illustrating the surface morphology and structural features of the nanocomposite; (e) Fourier-Transform Infrared (FT-IR) spectra highlighting the functional groups and chemical bonding within the composite; (f) X-Ray Diffraction (XRD) spectra confirming the crystalline phases and structural integrity of the MIL-53(Al)@BaTiO_3_ nanocomposite.

The present study employed a combination of advanced techniques, including Fourier-transform infrared spectroscopy (FT-IR), field-emission scanning electron microscopy (FE-SEM), scanning transmission electron microscopy (STEM), X-ray diffraction (XRD), and Brunauer–Emmett–Teller (BET) to characterize a composite metal-organic framework (MOF) BaTiO_3_ composite named MIL-53(Al)@BaTiO_3_. The morphological, and dimensional features of the hybrid MOF were examined using FE-SEM and STEM techniques. The resulting images of MIL-53(Al) and MIL-53(Al)@BaTiO_3_ nanocomposite material were shown in Figure 1(a, b, c, d). Both STEM and FESEM images revealed that the structure of MIL-53(Al) exhibits a nanoflower like structure. Conversely, the structure of the MIL-53(Al)@BaTiO_3_ composite was found to be spherical, as evidenced by the STEM and FESEM images.

The Fourier-transform infrared (FT-IR) spectrum of MIL-53(Al) and MIL-53(Al)@BaTiO_3_ nanocomposite material has been analyzed, and the results have been presented in Figure 1(e). The IR analysis confirms the presence of vibration bands around 1550 and 1430 cm^−1^, which are characteristic of the framework –(O–C–O)– groups, thereby confirming the existence of the dicarboxylate group. The absorption bands observed around 870 cm^−1^ in the FTIR spectra are attributed to the vibration bands of CO_3_^2−^ groups. The modes of vibration observed around 2990 cm^−1^ are assigned to the hydroxyl (–OH) group [26,27]. The absorption bands observed in the FTIR spectrum of MIL-53(Al)@BaTiO_3_ nanocomposite material confirm the formation of the desired structure.

The X-ray diffraction (XRD) pattern in Figure 1(f) for both pristine MIL-53(Al) and MIL-53(Al)@BaTiO_3_ is consistent with previous literature [28,29]. The crystal structure of the metal-organic framework (MOF), consisting of AlO_4_(OH)_2_ clusters linked by benzene dicarboxylate (BDC) bridging ligands, is shown by the distinctive peaks in the 2θ range of 5°–20°. The presence of the MIL-53(Al) signature peaks in the XRD patterns of MIL-53(Al)@BaTiO_3_ between 9°–20° confirms the crystallinity of MIL-53(Al). Additionally, distinctive peaks at 22.20°, 32.17°, 38.89°, 45.59°, 50.81°, 56.12°, and 65.79° can be seen in the BaTiO3 XRD pattern. According to the JCPDS 05–0626 standards, these peaks correlate to the (101), (110), (111), (200), (210), (211), and (220) planes of the cubic phase of BaTiO_3_ [29–31]. The results obtained confirm that the nanocomposite structure was successfully synthesized.

The sorption and desorption surface areas of the nanocomposites were found to be 8.968 m^2^g^−1^ and 12.6494 m^2^g^−1^, respectively, while the pore volumes were 0.311023 cm^3^g^−1^ and 0.344077 cm^3^g^−1^. These results indicate that the MIL-53(Al)@BaTiO_3_ nanocomposites possess high porosity and a significant surface-to-volume ratio.

### 3.2. Optimization studies

**Figure 2:**
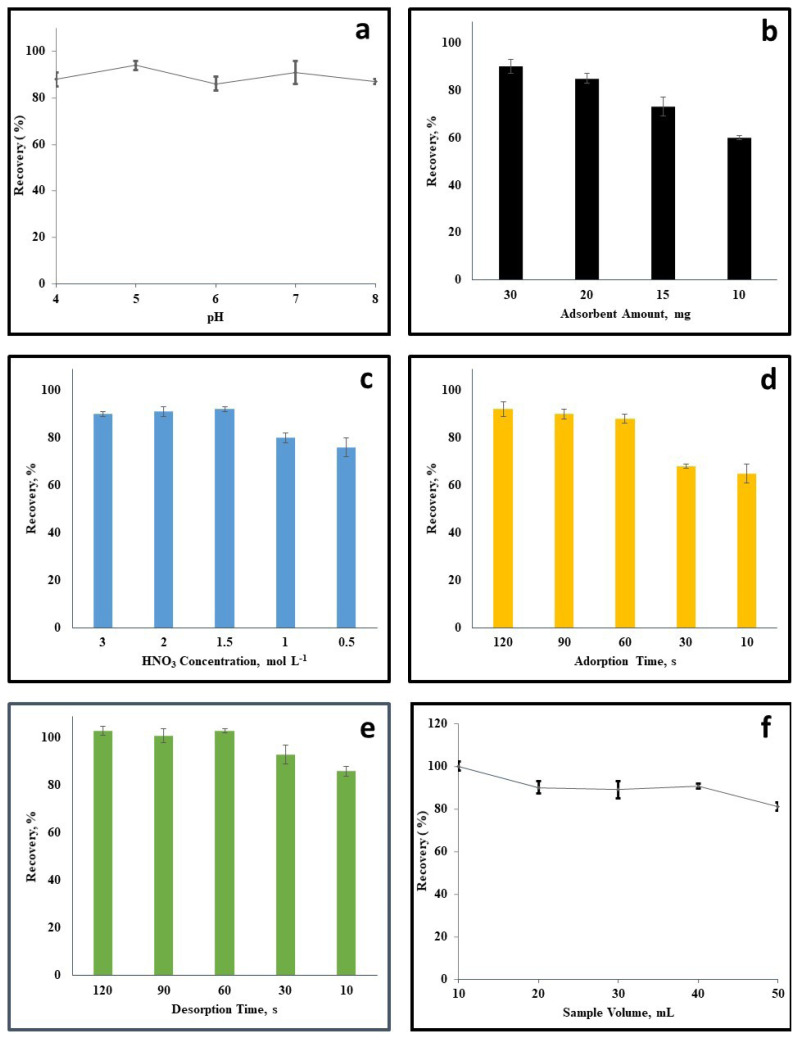
(a–f). The results of the optimization studies (pH, adsorbent amount, HNO_3_ concentration, adsorption time, desorption time and sample volume respectively). The dmSPE conditions were as follows: 40 mL sample solution containing Cd(II) (2.5 μg L^−1^); pH 5.0; 0.5 mL of 1.5 mol L^−1^ HNO_3_; 30 mg of MIL-53(Al)@BaTiO_3_ nanocomposite, n = 3.

The preconcentration of Cd (II) ions from seafood samples was studied, taking into account the different pH values of the buffers used, as the recovery rate is affected by the pH level [32,33]. The pH levels were adjusted between 4.0 and 8.0 using sodium acetate-acetic acid buffer (for pH 4.0 to 5.0) and phosphate-phosphoric acid buffer (for pH 5.0 to 8.0). Based on the results shown in Figure 2(a), it was determined that a pH level of 5.0 was the optimal for accurately determining the target element.

MIL-53(Al)@BaTiO_3_ nanocomposite is used in the suggested dmSPE technique to extract Cd(II) ions from seafood samples. For effective extraction, it is essential to identify the proper amount of sorbent. A range of 30 to 10 mg was investigated to determine the effect of adsorbent quantity on the highest possible extraction of Cd(II) (Figure 2(b)). The results indicated that using 30 mg of adsorbent led to the greatest recovery of Cd(II), which exceeded 90%. Therefore, the suggested dmSPE technique used 30 mg of MIL-53(Al)@BaTiO_3_ hybrid nanocomposite.

In order to effectively remove the Cd(II) sorbed on the MIL-53(Al)@BaTiO_3_ nanocomposite, a carefully selected desorption agent was utilized in this method. When choosing the appropriate desorption agent, it was important to consider various factors such as minimal boiling point, relatively low viscosity, and good solubility. The efficiency of the Cd(II) elution was assessed using various concentrations of HNO_3_, and the optimal results were achieved between 1.5 M HNO_3_ and 3 M HNO_3_, as depicted in Figure 2(c). In order to strike a balance between cost-effectiveness and environmental concerns, a total of 0.5 mL of 1.5 M HNO_3_ was selected as the optimum eluent.

To expedite the transfer of Cd(II) mass from seafood samples through the MIL-53(Al)@BaTiO_3_ nanocomposite surface in the dmSPE process, the solution was agitated using a vortex. The adsorption period was evaluated at various time intervals ranging from 2 min to ten s, with the goal of minimizing the duration of adsorption while ensuring sufficient interaction between the sorbent and the sample. Upon reaching the 90-s of the extraction period, the analytical signal in Figure 2(d) exhibited an enhancement. Conversely, periods shorter than 90 s yielded a decrease in signal. Therefore, it was determined that an optimal adsorption time of 90 s was necessary to achieve maximum efficiency.

The MIL-53(Al)@BaTiO_3_ material with Cd(II) was subjected to vortexing during the desorption process. To determine the optimal duration for desorption, a range of 10 s to 3 min was examined. Based on the data presented in Figure 2(e), it was observed that a quantitative point was reached after just 0.5 min of vortexing. Consequently, 0.5 min interval was deemed sufficient for removing Cd(II) from the sorbent surface, and was therefore chosen for further investigation.

In the dmSPE method, the sample volume plays a crucial role in obtaining a strong analyte response. For this study, various sample volumes ranging from 10 mL to 50 mL were tested, and it was found that a sample volume of 40 mL produced accurate quantitative analysis results (Figure 2f). However, a decrease in accuracy was observed at 50 mL. As a result, a sample volume of 40 mL was selected for further investigation.

### 3.3. Matrix effects

The presence of inorganic and organic substances in real-world samples can exert a pronounced effect on the analyte signal, especially in the instance of Cd. In order to assess the concentration of these interfering species, the study participants introduced various ions to a sample (2.5 μg L^−1^ Cd) and subsequently determined the effect on the Cd concentration. The results indicated that K^+^ and Cl^−^ could tolerate up to 40,000 times the amount of Cd (II) in the samples without importantly compromising the signals of the analyte, as confirmed by Table 1. Conversely, other ions, such as Zn^2+^ and Ni^2+^, displayed a tolerance limit of only up to 40 times. The researchers reached the opinion that coexisting ion levels in actual samples are unlikely to exceed these tolerance limits, and so would have no influence on Cd signals in the proposed approach. Eventually, the study highlights how important it is to detect interference from coexisting species in real-word samples and to establish these species’ tolerance limits to maintain the accuracy of the analytical technique.

### 3.4. Analytical features

Several parameters, including specificity, linearity, accuracy, sensitivity and linear range, can be employed to evaluate analytical figures. The LOD can be calculated by dividing three times the preconcentration calibration curve’s slope, or the standard deviation of 10 blank values, whereas the limit of quantification (LOQ) is determined by dividing ten times the standard deviation of ten blank readings by the slope of the calibration curve after preconcentration. The proposed method for Cd (II) achieved a good LOD and LOQ of 1.2μg^−^L^−1^ and 3.6 μgL^−1^, respectively. Precision describes how repeatable measures are, whereas accuracy describes how near the measured value is to the real amount. Under optimal circumstances, the analytical performance of the intended procedures is examined, and a satisfactory linear calibration curve (y = 12,517x − 0.0066) with an R^2^ of 0.9962 for Cd (II) at concentrations ranging from 3.9 to 250 μg L^−1^ is achieved. The RSD was calculated to be less than 5%. It was discovered that the maximum sample volume was 40mL. To ensure accurate results, 1.5M HNO_3_ was used as an eluent, resulting in quantitative values. The preconcentration factor, which is determined by dividing the sample’s final volume by the initial volume, was attained 80.

### 3.5. Applications

Bovine liver and seafood samples (consisting of shrimp, mussel, and tuna fish, each 0.25 g) were digested in a microwave oven with 5mL of HNO_3_ (65%) followed by 1mL of H_2_O_2_ (30%). The resulting mixture was filtered using filter paper and then diluted with ultra-pure water to a volume of 40mL. The optimized method described in Section 2.5 was used to analyze the digested samples for Cd (II) concentrations using HR-CS-FAAS. The validation of the presented dmSPE method was confirmed by analyzing two different Certified Reference Materials. The results are given in Table 2.

The recovery values were found to be quantitative. In addition to the dmSPE method, a standard addition method was also applied to analyze shrimp, mussel, and tuna fish samples, as shown in Table 3.

## 4. Conclusions

A novel, simple, and fast method for determining trace levels of Cadmium (Cd) with high accuracy and precision has been developed. The method leverages a new approach for synthesizing MIL-53(Al)@BaTiO_3_ nanocomposite. The synthesis is achieved using a microwave-assisted procedure, which takes 3 h, significantly reducing energy consumption and cost. The morphology and structure of the synthesized MIL-53(Al)@BaTiO_3_ was investigated using FE-SEM, STEM, FT-IR, BET, and XRD characterization tools. The synthesized material was utilized as an adsorbent in dispersive micro solid-phase extraction to determine Cd (II) using a High-Resolution Continuum Source-Flame Atomic Absorption Spectrometry (HR-CS-FAAS). Following the application of optimal conditions, the system’s LOD and LOQ values were determined to be 1.2 μg L^−1^ and 3.6 μg L^−1^, respectively. The recovery values ranged from 90% to 106%, demonstrating that the developed method is a suitable to other methods reported in the literature for determining Cd (II) at trace levels from seafoods. This method exhibits a high preconcentration factor and low LOD and LOQ, resulting in sensitive and accurate analysis of complex matrices such as seafoods (Table 4).

## Figures and Tables

**Table 1:** Effects of some interfering species on Cd(II) after separation/preconcentration with dmSPE, n = 3.

Interfering Ions	Added As	Concentration, mg L^−1^	Recovery, %
**Na** **^+^**	Na_2_SO_4_	500	100 ± 2
**K** **^+^**	KCl	1000	101 ± 4
**Fe** **^3+^**	FeCl_3_.6H_2_O	5	95 ± 1
**Mn** **^2+^**	Mn(NO_3_)_2_·4H_2_O	5	103 ± 5
**Co** **^2+^**	Co(NO_3_)_2_·6H_2_O	2	95 ± 3
**Zn** **^2+^**	Zn(NO_3_)_2_·6H_2_O	1	95± 2
**Ni** **^2+^**	Ni(NO_3_)_2_·6H_2_O	1	89 ± 4
**Cl** **^−^**	KCl	1000	101 ± 4
**SO** **_4_** **^2−^**	Na_2_SO4	1040	100 ± 2
**NO** **_3_** **^−^**	NaNO_3_	400	95 ± 5

**Table 2:** Application of method to certified reference materials, n = 3.

CRM	Certificate Value	Found	Recovery, %
**SPS-WW2 (μg L** **^−1^** **)**	100	92.6 ± 4	93
**NIST CRM 1577b (μg g** **^−1^** **)**	0.5	0.48 ± 0.03	96

SPS-WW2: wastewater, NIST CRM 1577b: bovine liver.

**Table 3:** The addition-recovery tests the dmSPE technique to detect Cd(II) in real samples, n = 3.

Sample	Added, μg g^−1^	Found, μg g^−1^	Recovery, %
**Shrimp**	0	0.03 ± 0.01	-
	0.4	0.39 ± 0.02	90
	0.8	0.82 ± 0.02	96
	1.6	1.64 ± 0.04	101
**Mussel**	0	0.07 ± 0.01	-
	0.4	0.46 ± 0.01	98
	0.8	0.87 ± 0.04	100
	1.6	1.77 ± 0.06	106
**Tuna Fish**	0	UDL	
	0.4	0.42 ± 0.01	105
	0.8	0.76 ± 0.03	95
	1.6	1.63 ± 0.03	102

UDL: Under detection limit

**Table 4:** Comparison of the dmSPE method with the other methods in the literature.

Extraction method	Adsorbent	Instrument	Sample Matrix	LOD (μg L^−1^)	EF/PF	RSD (%)	References
**VAdSP-μE**	MPCP	FAAS	Water and Food	1.7	50	0.7	[34]
**Um-SPμE**	8 -HQ embedded benzophenone	FAAS	Coal Gangue Soil	0.065	80.4	4.5	[35]
**VA-dSPμE**	PvbXa	FAAS	Water and Food	0.06	160	4.3	[36]
**SPE**	GO-ZnO	FAAS	Hibiscus Tea	3.83	8	-	[37]
**UA-D-μSPE**	Fe_3_O_4_@HPMC	FAAS	Food	0.4	50	<4.2	[38]
**D-μ-SPE**	Fe_3_O_4_/ZnO@SNC	FAAS	Water	0.4	51.1	1.5	[39]
**MF-MNP-DSPE**	MnFe_2_O4	FAAS	Lake Water	1.3	135	-	[40]
**dmSPE**	MIL-53(Al)@BaTiO_3_	HR-CS-FAAS	Seafood and Water	1.2	80	<5.0	Present study

**VAdSP-μE**: Vortex-assisted dispersive solid-phase microextraction, MPCP: Magnetic polystyrene-b- poly dimethyl siloxane hydrophobic block copolymer, **Um-SPμE**: Ultrasound assisted modified solid phase micro-extraction, **VA-dSPμE**: Vortex-assisted dispersive solid-phase microextraction, **PvbXa**: Polyvinyl benzyl xanthate, **SPE**: Solid phase extraction, **UA-D-μSPE**: Ultrasound-assisted dispersive microsolid-phase extraction, **D-μ-SPE**: Dispersive micro-solid-phase extraction

## References

[1] Kaçar E Heavy metal concentrations in various tissues of two fish species from Damsa Dam Lake (Turkey) and associated health risk assessment Journal of Trace Elements in Medicine and Biology 2024 81 127339 10.1016/j.jtemb.2023.127339 37976961

[2] Dahaji RRM Moghimi A Shahbaz H Faraji H Azizinejad F Preparation of modified multiwalled carbon nanotubes (L-Arg-CS/MWCNTs-COOH/Fe_3_O_4_) as sorbent for dispersive solid phase extraction of Cu(II), Pb(II) and Cd(II) in wastewaer samples Revue Roumaine De Chimie 2023 68 495 505 10.33224/rrch.2023.68.10-12.01

[3] Kurnaz Yetim N Berberoğlu EA Aslan N Koç MM Özcan C Sonochemical removal of Pb (II) ions from the water medium using Bi2S3 nanostructres International Journal of Environmental Analytical Chemistry 2024 104 3586 3601 10.1080/03067319.2022.2088288

[4] Conchado-Amado P Sánchez-Piñero J Moreda-Piñeiro J Turnes-Carou I López-Mahía P Ultra-trace mercury determination in seawater after vortex-assisted liquid-liquid micro-extraction Spectrochimica Acta Part B: Atomic Spectroscopy 2023 204 106683 10.1016/j.sab.2023.106683

[5] Khan M Ozalp O Khan M Soylak M Fe_3_O_4_-Ti_3_AlC_2_ max phase impregnated with 2-(5-Bromo-2-pyridylazo-5-(diethylamino) phenol for magnetic solid phase extraction of Cadmium, lead and cobalt from water and food samples Journal of Molecular Liquids 2022 368 120685 10.1016/j.molliq.2022.120685

[6] Smirnova SV Ilin DV Pletnev IV Extraction and ICP-OES determination of heavy metals using tetrabutylammonium bromide aqueous biphasic system and oleophilic collector Talanta 2021 221 121485 10.1016/j.talanta.2020.121485 33076095

[7] Khadhar S Sdiri A Chekirben A Azouzi R Charef A Integration of sequential extraction, chemical analysis and statistical tools for the availability risk assessment of heavy metals in sludge amended soils Environmental Pollution 2020 263 114543 10.1016/j.envpol.2020.114543

[8] Khan WA Arain MB Soylak M Nanomaterials-based solid phase extraction and solid phase microextraction for heavy metals food toxicity Food and Chemical Toxicology 2020 145 111704 10.1016/j.fct.2020.111704 32853698

[9] Çıtlakoğlu M Yolcu Z Synthesis and characterization of zinc(II) methacrylate monomer complex and adsorption properties of zinc(II) ion-imprinted polymer Inorganica Chimica Acta 2023 555 121605 10.1016/j.ica.2023.121605

[10] Charkiewicz AE Omeljaniuk WJ Nowak K Garley M Nikliński J Cadmium Toxicity and Health Effects—A Brief Summary Molecules 2023 28 6620 10.3390/molecules28186620 37764397 PMC10537762

[11] Wang M Chen Z Song W Hong D Huang L A review on Cadmium Exposure in the Population and Intervention Strategies Against Cadmium Toxicity Bulletin of Environmental Contamination and Toxicology 2021 106 65 74 10.1007/s00128-020-03088-1 33486543

[12] Cui C He M Chen B Hu B γ-mercaptopropyltrimethoxysilane Coated Stir Bar Sorptive Extraction Combined with Inductively Coupled Plasma Mass Spectrometry for the Determination of Copper, Cadmium and Mercury in Environmental Water Samples Atomic Spectroscopy 2023 44 440 447 10.46770/AS.2023.291

[13] Akdogan A Divrikli U Soylak M Elçi L Assessment of heavy metal levels in street dust samples from Denizli, Turkey, and analysis by flame atomic absorption spectrometry Atomic Spectroscopy 2016 37 25 29 10.46770/AS.2016.01.005

[14] Aladaghlo Z Malekzadeh S Sahragard A Fakhari AR Synthesis of Fe_3_O_4_@SiO_2_-creatine as a new nanosorbent for dispersive magnetic solid-phase microextraction of copper ions from water, food, and soil samples Journal of Food Composition and Analysis 2024 129 106097 10.1016/j.jfca.2024.106097

[15] Bayrak HE Bulut VN Tüfekçi M Bayrak H Duran C Comparative study for the separation, preconcentration, and determination of copper and cadmium in real samples by using two different ligands Turkish Journal of Chemistry 2016 40 93 105 10.3906/kim-1505-11

[16] Sajid M Nazal MK Ihsanullah I Novel materials for dispersive (micro) solid-phase extraction of polycyclic aromatic hydrocarbons in environmental water samples: A review Analytica Chimica Acta 2021 1141 246 262 10.1016/j.aca.2020.07.064 33248658

[17] Ghorbani M Aghamohammadhassan M Ghorbani H Zahibi A Trends in sorbent development for dispersive micro-solid phase extraction Microchemical Journal 2020 158 105250 10.1016/j.microc.2020.105250

[18] Bahçıvan A Şaylan M Sagdic O Bakırdere S CoSn(OH)_6_ nanocubes as a solid sorbent for the effective preconcentration of copper ions in cinnamon (Cinnamomum zeylanicum) extract Food Chemistry 2024 447 139037 10.1016/j.foodchem.2024.139037 38513484

[19] Li XD Xin L Rong WT Liu XY Deng WA Effect of heavy metals pollution on the composition and diversity of the intestinal microbial community of a pygmy grasshopper (Eucriotettix oculatus) Ecotoxicology and Environmental Safety 2021 223 112582 10.1016/j.ecoenv.2021.112582 34365209

[20] Liu C Wang J Wan J Yu C MOF-on-MOF hybrids: Synthesis and applications Coordination Chemistry Reviews 2021 432 213743 10.1016/j.ccr.2020.213743

[21] Uflyand IE Zhinzhilo VA Nikolaevskaya VO Kharisov BI González CMO Recent strategies to improve MOF performance in solid phase extraction of organic dyes Microchemical Journal 2021 168 106387 10.1016/j.microc.2021.106387

[22] Moinfar S Khodayari A Sohrabnezhad S Aghaei A Jamil LA MIL-53(Al)/Fe_2_O_3_ nanocomposite for solid-phase microextraction of organophosphorus pesticides followed by GC-MS analysis Microchimica Acta 2020 187 1 10 10.1007/s00604-020-04621-z 33165626

[23] Kori AH Uzcan F Soylak M BaTiO_3_ is a novel adsorbent for solid-phase extraction of copper at trace levels in food and water samples before HR-CS-FAAS detection Journal of Food Composition and Analysis 2023 122 105474 10.1016/j.jfca.2023.105474

[24] Zhang C Miao J Wang Z Yang G Liu X Dielectric and Ferroelectric Properties of In Situ Synthesized Co(Fe1−xMnx)_2_O_4_/BaTiO_3_ Composite Ceramics Journal of Electronic Materials 2022 51 6286 6296 10.1007/s11664-022-09820-3

[25] Sun L Yin M Li Z Tang S Facile microwave-assisted solvothermal synthesis of rod-like aluminum terephthalate [MIL-53(Al)] for CO_2_ adsorption Journal of Industrial and Engineering Chemistry 2022 112 279 286 10.1016/j.jiec.2022.05.021

[26] Do XD Hoang VT Kaliaguine S MIL-53 (Al) mesostructured metal-organic frameworks Microporous and Mesoporous Materials 2011 141 135 139 10.1016/j.micromeso.2010.07.024

[27] Bhuiyan MR Alam MM Momin MA Uddin MJ Islam M Synthesis and Characterization of Barium Titanate (BaTiO_3_) Nanoparticle International Journal of Mechanical Engineering and Technology 2012 1 21 24

[28] Amorntep N Namvong A Wongsinlatam W Remsungnen T Siritaratiwat A Electrical performance enhancement of a triboelectric nanogenerator based on epoxy resin/BaTiO_3_ by Al nanopowder addition for low power electronic devices Nanotechnology 2023 34 425401 10.1088/1361-6528/ace724 37526494

[29] Wang F Mao H Chen X Li W Liu Z Modification of BaTiO_3_ for diversified applications by single Nd element substitution with wide doping range Journal of Materials Science: Materials in Electronics 2023 34 1062 10.1007/s10854-023-10482-y

[30] Karami A Shomal R Sabouni R Murtaza SZM Ghommem M Photocatalytic degradation of diclofenac using hybrid MIL-53 (Al)@TiO_2_ and MIL-53 (Al)@ZnO catalysts The Canadian Journal of Chemical Engineering 2023 101 2660 2676 10.1002/cjce.24666

[31] Wang W Cao L Liu W Su G Zhang W Low-temperature synthesis of BaTiO_3_ powders by the sol-gel-hydrothermal method Ceramics International 2013 39 7127 7134 10.1016/j.ceramint.2013.02.055

[32] Efe E Imamoglu M Solid-phase extraction (SPE) of Ni(II), Cd(II) and Pb(II) ions using pyromellitic dianhydride functionalized silica gel prior to flame atomic absorption spectrometric determination Desalination and Water Treatment 2024 317 100158 10.1016/j.dwt.2024.100158

[33] Soylak M Maulana R Ultrasound assisted magnetic solid phase extraction of copper(II) and lead(II) in environmental samples on Magnetic Activated Carbon Cloth International Journal of Environmental Analytical Chemistry 2023 103 2542 2554 10.1080/03067319.2021.1895136

[34] Yılmaz S Ullah N Citak D Hazer B Tuzen M Vortex-assisted dispersive solid-phase microextraction of cadmium and copper on magnetic polystyrene-b-poly dimethyl siloxane hydrophobic block copolymer for their atomic absorption spectrometric determination in water, soft drink and food samples Journal of Food Composition and Analysis 2023 123 105487 10.1016/j.jfca.2023.105487

[35] Lashari AA Kazi TG Baig JA Afridi HI Lashari A An ultrasound assisted modified solid phase micro-extraction technique for enrichment of cadmium and lead in aqueous extract of coal gangue soil samples Geoderma 2023 437 116601 10.1016/j.geoderma.2023.116601

[36] Altunay N Hazer B Lanjwani MF Tuzen M Ultra-Sensitive Determination of Cadmium in Food and Water by Flame-AAS after a New Polyvinyl Benzyl Xanthate as an Adsorbent Based Vortex Assisted Dispersive Solid-Phase Microextraction: Multivariate Optimization Foods 2023 12 3620 10.3390/foods12193620 37835273 PMC10572459

[37] Demir C Öner M Çetin G Bakırdere S A simple and effective graphene oxide-zinc oxide nanocomposite based solid phase extraction method for the determination of cadmium in tea matrix at trace levels by flame atomic absorption spectrophotometry Journal of Food Composition and Analysis 2024 127 105968 10.1016/j.jfca.2024.105968

[38] Esmaeili Lashkarian E Ahmadi S Beigmohammadi F Ultrasound-Assisted Dispersive Magnetic Solid-Phase Extraction Using Fe_3_O_4_@Hydroxypropyl Methylcellulose Combined with Flame Atomic Absorption Spectrometry for Determination of Cadmium(II) in Food Samples Arabian Journal for Science and Engineering 2023 49 209 219 10.1007/s13369-023-08029-8

[39] Tadese M Amde M Leta Danno B Benti L Megersa N Magnetised and sulfide functionalised zinc oxide nanocomposite for dispersive micro-solid-phase extraction of lead (II) and cadmium (II) in water samples International Journal of Environmental Analytical Chemistry 2023 103 1203 1221 10.1080/03067319.2020.1871476

[40] Karaman DN Serbest H Kilinç Y Demirel R Bakırdere S Trace cadmium determination in lake water matrix by flame atomic absorption spectrometry after manganese ferrite magnetic nanoparticles-based dispersive solid phase extraction Clean-Soil, Air, Water 2023 51 2200186 10.1002/clen.202200186

